# Imaging Mass Spectrometry Revealed the Accumulation Characteristics of the 2-Nitroimidazole-Based Agent “Pimonidazole” in Hypoxia

**DOI:** 10.1371/journal.pone.0161639

**Published:** 2016-08-31

**Authors:** Yukiko Masaki, Yoichi Shimizu, Takeshi Yoshioka, Fei Feng, Songji Zhao, Kenichi Higashino, Yoshito Numata, Yuji Kuge

**Affiliations:** 1 Shionogi Innovation Center for Drug Discovery, Discovery Research Laboratory for Innovative Frontier Medicines, Shionogi & Co., Ltd., Sapporo, Japan; 2 Faculty of Pharmaceutical Sciences, Hokkaido University, Sapporo, Japan; 3 Central Institute of Isotope Science, Hokkaido University, Sapporo, Japan; 4 Graduate School of Medicine, Hokkaido University, Sapporo, Japan; University of Nebraska Medical Center, UNITED STATES

## Abstract

Hypoxia, or low oxygen concentration, is a key factor promoting tumor progression and angiogenesis and resistance of cancer to radiotherapy and chemotherapy. 2-Nitroimidazole-based agents have been widely used in pathological and nuclear medicine examinations to detect hypoxic regions in tumors; in particular, pimonidazole is used for histochemical staining of hypoxic regions. It is considered to accumulate in hypoxic cells via covalent binding with macromolecules or by forming reductive metabolites after reduction of its nitro group. However, the detailed mechanism of its accumulation remains unknown. In this study, we investigated the accumulation mechanism of pimonidazole in hypoxic tumor tissues in a mouse model by mass spectrometric analyses including imaging mass spectrometry (IMS). Pimonidazole and its reductive metabolites were observed in the tumor tissues. However, their locations in the tumor sections were not similar to the positively stained areas in pimonidazole-immunohistochemistry, an area considered hypoxic. The glutathione conjugate of reduced pimonidazole, a low-molecular-weight metabolite of pimonidazole, was found in tumor tissues by LC-MS analysis, and our IMS study determined that the intratumor localization of the glutathione conjugate was consistent with the area positively immunostained for pimonidazole. We also found complementary localization of the glutathione conjugate and reduced glutathione (GSH), implying that formation of the glutathione conjugate occurred in the tumor tissue. These results suggest that in hypoxic tumor cells, pimonidazole is reduced at its nitro group, followed by conjugation with GSH.

## Introduction

Hypoxia (low oxygen concentration) in tumors contributes to promoting tumor progression and angiogenesis, and has negative effects on their response to radiotherapy and chemotherapy [1, 2]. Therefore, the ability to identify early the location and extent of hypoxia can provide useful information for determining the therapeutic strategy, which has implications for the clinical outcome of the cancer.

To detect hypoxic regions in tissues, various kinds of imaging probes have been developed for immunohistochemistry, and also for molecular imaging (including nuclear imaging and fluorescence imaging). Most of these probes contain a 2-nitroimidazole structure, because 2-nitroimidazole undergoes reductive metabolism, which leads to its specific accumulation in hypoxic areas [3].

Pimonidazole is a 2-nitroimidazole-based exogenous hypoxia marker extensively used for measuring tumor hypoxia by immunohistochemical staining in pathological research. Pimonidazole, as for other 2-nitroimidazole-based agents, is believed to bind covalently to macromolecules in hypoxic cells after reduction of the nitro group of its imidazole ring [4]. However, the detailed mechanism of its accumulation remains unknown.

Histological analyses such as immunohistochemistry and nuclear imaging including autoradiography (ARG) and positron emission tomography (PET) using radiolabeled agents cannot distinguish the distributions of metabolites of the agent from the unchanged agent within tissues [5, 6]. Liquid chromatography-tandem mass spectrometry (LC-MS/MS) cannot offer spatial information on the drug distribution within tissues [7]. Therefore, these conventional analytical techniques cannot provide chemical/structural information and spatial information in the same experiment, which makes it hard to determine the detailed mechanism of pimonidazole accumulation in hypoxic regions.

Imaging mass spectrometry (IMS) is a novel imaging technique that can directly visualize the distribution of specific molecules in tissue sections based on their molecular masses [8]. Over the past few years, this technique has been widely used to investigate the distributions of a variety of biomolecules, for example peptides, lipids, drugs and endogenous metabolites [9–11]. IMS can evaluate the distributions of numerous molecules in a single measurement without a specialized probe because of its MS-based detection. This property enables it to distinguish distributions of metabolites of a drug from unchanged drug in tissue sections [12]. IMS should be an effective imaging technique for distribution measurements of each specific drug-derived metabolite.

Recently, we elucidated the mechanism of ^18^F-fluoromisonidazole (FMISO) accumulation in hypoxic tumor tissues using IMS combined with radioisotope analysis [13]. ^18^F-FMISO, an ^18^F-labeled 2-nitroimidazole derivative, is the most widely used hypoxia-imaging probe for PET diagnosis. In that study, we demonstrated that most of the radioactivity derived from ^18^F-FMISO was present in low-molecular-weight substances in hypoxic tumors. This was in contrast to observations made with conventional views of FMISO covalent binding to macromolecules, and a glutathione conjugate of reduced FMISO (amino-FMISO) was a component responsible for FMISO accumulation in the hypoxic regions of tumors [13]. This result suggests that ^18^F-FMISO incorporated in hypoxic cells is conjugated with glutathione following reduction of its nitro group, and thus the radioactivity was trapped in the cells. However, it is still unclear whether the accumulation mechanism mentioned above is a common phenomenon among 2-nitroimidazole-based agents including pimonidazole, or if it is only seen with FMISO.

To reveal the detailed accumulation mechanism of pimonidazole in hypoxic tumor tissues, we performed an IMS study which visualized the distribution of pimonidazole and its low-molecular-weight metabolites, and compared the images with pimonidazole-immunohistochemistry, a conventional analysis of pimonidazole distribution. Furthermore, we also compared the distributions of pimonidazole metabolites and endogenous molecules that might be involved in the metabolism of pimonidazole.

## Materials and Methods

### Chemicals and reagents

All chemicals were commercially available and of the highest purity. Pimonidazole (Hypoxyprobe-1) was purchased from HPI Inc. (Burlington, MA, USA). HPLC-grade methanol and acetonitrile were purchased from Kanto Chemical Co., Inc. (Tokyo, Japan). Trifluoroacetic acid (TFA), ammonium hydrogen carbonate and the reduced form of glutathione were from Wako Pure Chemical Co., Ltd. (Osaka, Japan). The oxidized form of glutathione was from Nacalai Tesque, Inc. (Kyoto, Japan). 2,5-Dihydroxybenzioc acid (DHB) and indium tin oxide (ITO) glass slides were purchased from Bruker Daltonics (Billerica, MA, USA). Complete protease inhibitor cocktail was from Roche Diagnostics (Basel, Switzerland).

### Synthesis of glutathione conjugate of amino-pimonidazole

Glutathione conjugate of amino-pimonidazole was synthesized by reference to previous reports [14, 15]. Briefly, pimonidazole (22 mg) was dissolved in 200 ml water. After the solution was heated to 55°C, 76 mg ammonium chloride and 48 mg zinc were added and the mixture was refluxed for 2 h. After filtration of the mixture to remove the zinc, reduced glutathione (GSH) (618.4 mg/200 ml in 0.05 M phosphate buffer (pH 7.0)) was added and heated at 37°C overnight. The reaction mixture was evaporated and then purified by reversed-phase HPLC to obtain the glutathione conjugate of amino-pimonidazole (2.6 mg, 6.5%) using a Shimadzu-HPLC gradient system (LC-10AD; Shimadzu Corporation, Kyoto, Japan) equipped with an Atlantis T3 column (250 mm × 19 mm, 5 μm, Waters Co., Milford, MA, USA). Chromatographic separation was achieved by water/acetonitrile (97:3) containing 0.05% TFA. The total HPLC run time was 40 min at a flow rate of 17 ml/min.

^1^H NMR (D_2_O) δ6.98 (s, 1H), 4.46 (m, 1H), 4.29 (m, 1H), 3.82 (d, 2H, J = 8.0), 3.76 (t, 1H, J = 6.0), 3.58 (m, 1H), 3.53 (m, 1H), 3.46 (dd, 1H, J = 14.5, 3.0), 3.32 (dd, 1H, J = 14.5, 7.0), 3.24 (m, 2H), 3.15 (m, 1H), 3.04 (m, 1H), 3.03 (m, 1H), 2.93 (m, 1H), 2.52 (m, 2H), 2.14 (m, 2H), 1.91 (m, 2H), 1.77 (m, 2H), 1.48 (m, 1H), 1.26 (m, 1H). High-resolution (HR) MS (m/z) (ESI, pos): [M+H]^+^ calcd. for C_21_H_35_N_7_O_7_S 530.23914; found, 530.24023.

### Tumor xenograft model

All experimental protocols were approved by the Laboratory Animal Care and Use Committee of Hokkaido University and performed in accordance with the Guidelines for Animal Experiments at the Graduate School of Medicine, Hokkaido University. Nine-week-old male BALB/c athymic nude mice (Japan SLC, Inc., Hamamatsu, Japan) were housed in groups (4–5 animals) in ventilated cages. The environmental conditions were: temperature ~25°C, 40%–70% humidity, and a 12-h light/12-h dark cycle. The mice were fed a standard laboratory diet (FR-1, Sankyo Laboratory Service Corporation, Tokyo, Japan). Food and water were supplied ad libitum. A human head and neck cancer xenograft model was established using the human head and neck cancer cell line FaDu (American Type Culture Collection, Manassas, VA, USA). This cell line is an established human hypopharyngeal squamous cell carcinoma that grows as an undifferentiated carcinoma in nude mice [16]. The FaDu cells were maintained in Eagle’s Minimum Essential Medium (Sigma-Aldrich, St Louis, MO, USA) supplemented with 10% fetal bovine serum and penicillin (100 U/ml)–streptomycin (100 μg/ml) at 37°C in a humidified atmosphere of 95% air and 5% CO_2_. FaDu cells (5×10^6^ cells) suspended in 100 μl phosphate-buffered saline were inoculated subcutaneously into the right flank of each mouse. Further experiments were performed after a 2-week tumor growth period. At that time, the body weights were 24.0 ± 1.2 g, and the tumor volumes were 853 ± 612 mm^3^, calculated using the formula: π/6 × largest diameter × (smallest diameter)^2^. Mice were monitored for health and body condition every 3–4 days and observed not to show any abnormal symptoms such as weight loss or injury. Mice with tumor sizes of largest diameter ≥2 cm were excluded from this study. Excluded animals were euthanized by exsanguination under anesthesia with 1.5%–2.0% isoflurane. All animal manipulations were performed using sterile techniques.

### Animal experiments

Pimonidazole (200 mg/kg) dissolved in saline was injected into the tumor-bearing mice via the tail vein. Mice were sacrificed at 0.5, 2 and 4 h after administration by inhalation anesthesia with 1.5%–2.0% isoflurane. Tumor tissues were immediately excised and frozen in dry ice powder. Serial cross sections of 10-μm thickness were immediately cut and thaw-mounted on glass slides using a CM3050-cryostat (Leica Microsystems; Wetzlar, Germany).

### Immunohistochemical staining of pimonidazole

Tumor sections mounted on immunocoat micro slides (Muto Pure Chemicals Co., Ltd., Tokyo, Japan) were used for the immunohistochemical staining of pimonidazole to assess hypoxic areas in tumor tissue. After rehydration, endogenous peroxidase activity was blocked for 10 min with 0.3% hydrogen peroxide. The slides were incubated with a monoclonal antibody to pimonidazole (HPI Inc., Burlington, BA, USA) for 30 min at 37°C, followed by incubation with biotin-conjugated F(ab′)_2_ for 15 min at 37°C. The bound antibody complex was then visualized by incubation with streptavidin and 3,3′-diaminobenzidine tetrahydrochloride. The images of the tumor sections stained by the anti-pimonidazole antibody were captured under a microscope (Biozero BZ-8000; Keyence Co., Osaka, Japan).

### Sample preparation for MALDI-IMS

The tumor sections were placed onto indium tin oxide-coated glass slides and stored at −80°C until analysis. Prior to matrix coating and mass spectrometric analysis, slides were placed in a vacuum desiccator for 15 min at room temperature and optical images were acquired using a scanner (GT-X820; Seiko Epson Corporation, Nagano, Japan) to identify the location of the tissues. Sections were then coated with the matrix solution (30 mg/ml DHB dissolved in 1:1 (v/v) methanol–water containing 0.2% TFA) using an ImagePrep™ automated device using vibrational vaporization technology (Bruker Daltonics Inc., Billerica, MA, USA). For evaluation of the contribution of low-molecular-weight metabolites to immunostained areas of pimonidazole, the serial sections were washed with 50% ethanol and 100% ethanol twice and once, respectively, to remove pimonidazole and its derivatives that were not covalently conjugated to macromolecules. After drying the sections, matrix coating was conducted by the same method described above.

### MALDI-IMS study

IMS analysis was performed using a 7T Bruker solariX XR MALDI FT-ICR MS (Bruker Daltonics Inc.) equipped with a SmartBeam II UV laser. Mass spectra were analyzed and obtained using data analysis software (Bruker Daltonics Inc.). After acquisition of mass spectra, FlexImaging software (Bruker Daltonics Inc.) was used for data processing and image generation. All imaging data were normalized by total ion current (TIC). The laser energy and the raster step size were set at 30%–70% and 125 μm, respectively. Analytes were detected in the positive-ion mode. Mass peaks were assigned to each metabolite using exact mass values with a mass tolerance of 0.005. The obtained peak was accumulated and split by collision-induced dissociation (CID)-fragmentation to obtain structural information. Reproducibility was confirmed by examining tumor sections from four pimonidazole-treated mice at each timepoint. We also show the specificity of the glutathione conjugate in pimonidazole-treated mice compared with pimonidazole-untreated (control) mice (S3 Fig). We did not perform further statistical analyses in this study. In this study, 80% and 0% of the highest intensity normalized by TIC were set as the maximum and minimum of the intensity scale, respectively.

### LC-MS analysis

The tumor of each mouse was weighed, suspended in PBS with protease inhibitor cocktail (Roche Diagnostics Ltd., Mannheim, Germany) (4 ml/g of tissue) and then crushed at 4,000 rpm for 1 min with 3- and 5-mm-diameter zirconia beads using a Micro Smash™ device (Tomy Seiko Co., Ltd., Tokyo, Japan) at 4°C. The homogenized samples were extracted with methanol.

To evaluate the production of the glutathione conjugate of amino-pimonidazole in the tumor homogenate, the sample was injected into the LC-MS system. A Shimadzu-HPLC gradient system (LC-20AD system; Shimadzu Corporation) with 7T Bruker solariX XR ESI FT-ICR MS was used for LC-MS analysis. The LC-MS system was controlled by Hystar 4.0 software (Bruker Daltonics Inc.). Chromatographic separation was carried out using a YMC-Triart C18 column (150 mm × 4.6 mm, 3 μm; YMC Co., Ltd., Kyoto, Japan) at 40°C. Chromatographic separation was achieved using a mobile phase composed of 15 mM ammonium hydrogen carbonate and acetonitrile in the ratio 91:9. The total UPLC run time was 7 min. The flow rate was 1 ml/min. The eluate was split in a ratio of 1:5, of which 0.2 ml/min was directed to the mass spectrometer and the reminder to waste. ESI was performed in the positive-ion mode.

## Results

### Distribution of pimonidazole and its derivatives in tumors

**Fig 1:**
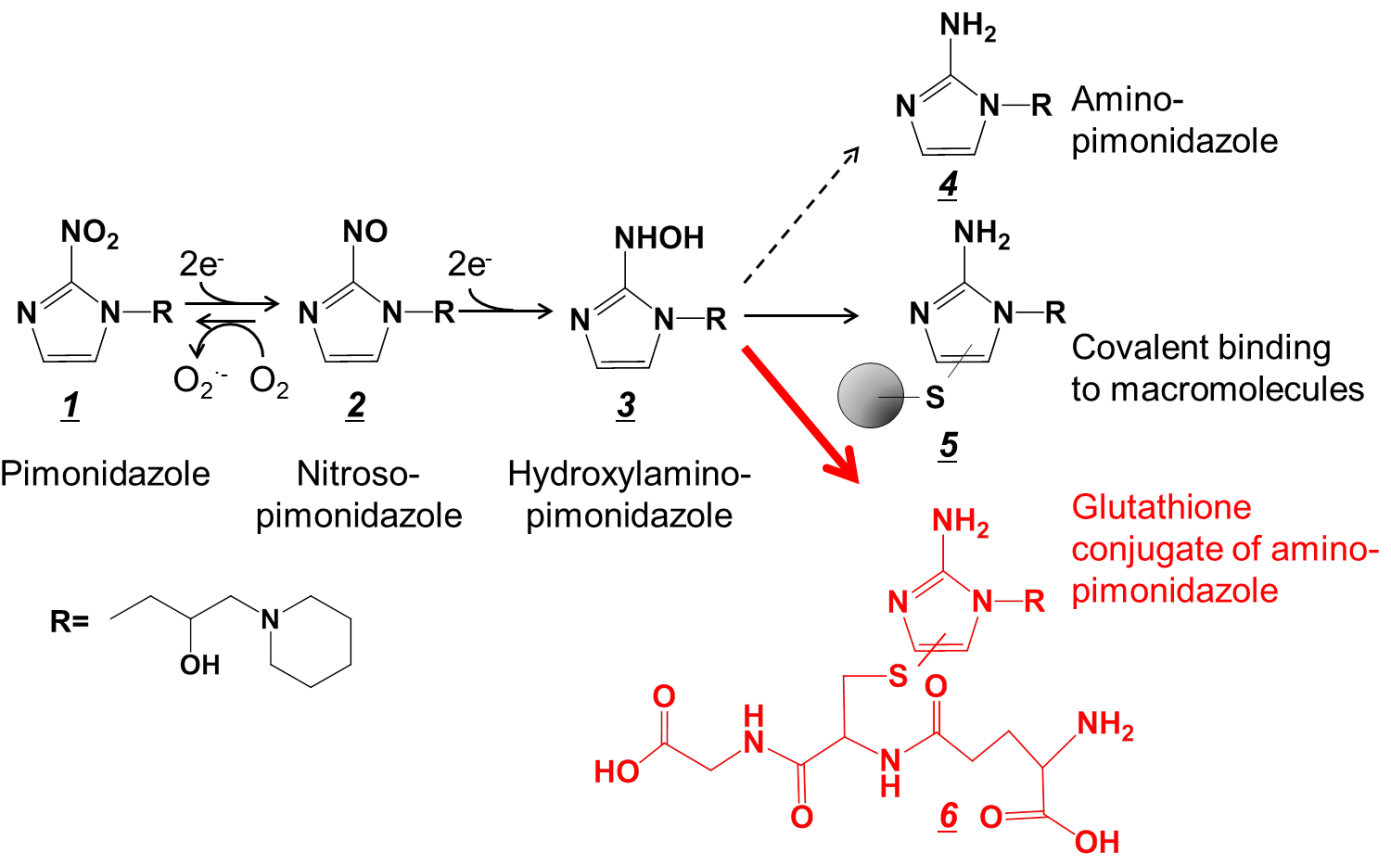
Proposed mechanism of accumulation of pimonidazole in hypoxic tissue regions.

**Fig 2:**
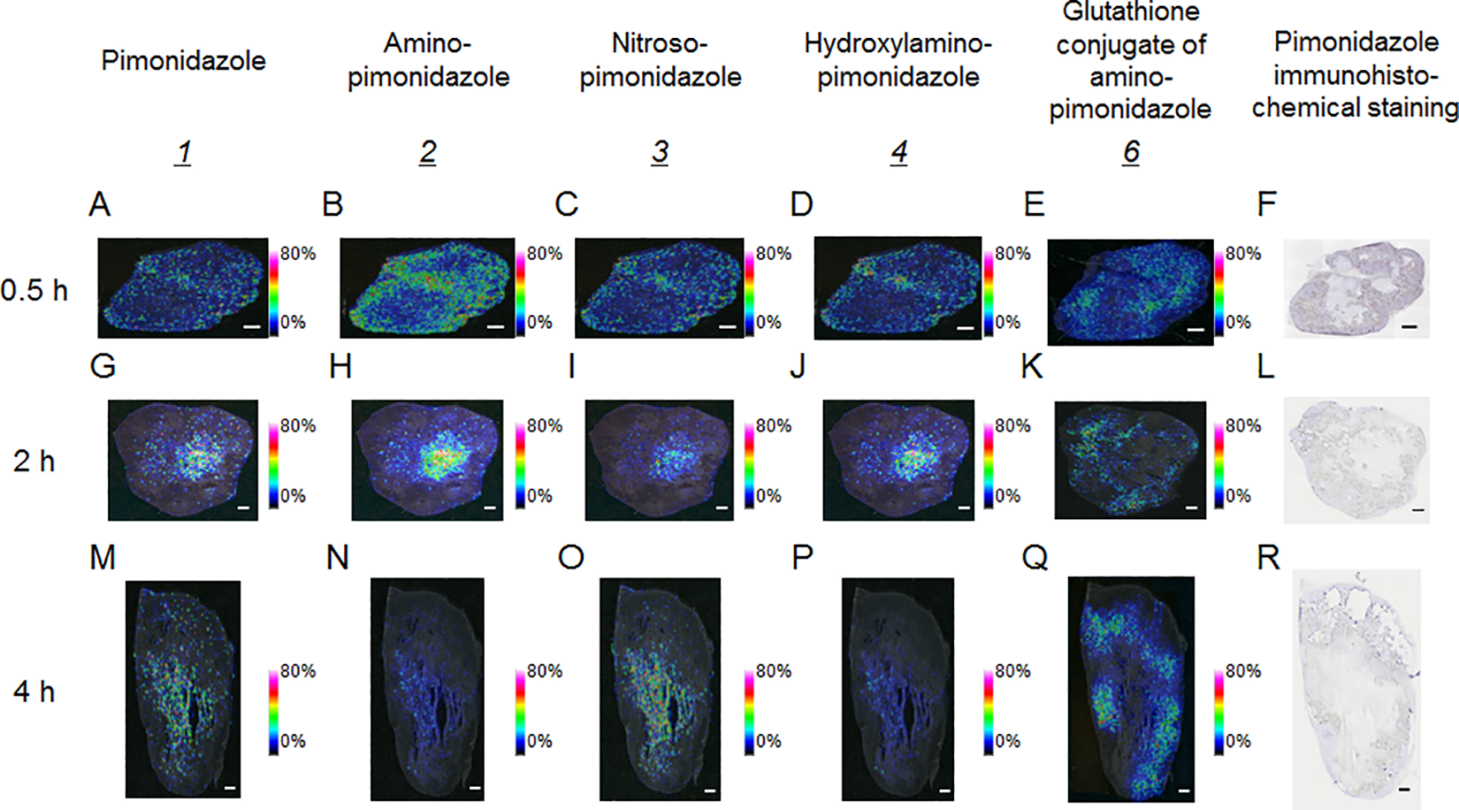
Representative mass spectrometric images of pimonidazole and its low-molecular mass metabolites and pimonidazole immunohistochemical staining in mouse tumors 0.5, 2 and 4 h after administration of pimonidazole. The scale bar represents 1 mm. (A)–(F): Mass spectrometric images of mouse tumor 0.5 h after administration. (G)–(L): Mass spectrometric images of mouse tumor 2 h after administration. (M)–(R): Mass spectrometric images of mouse tumor 4 h after administration. (A), (G), (M): Mass spectrometric images of m/z 255.145, representing pimonidazole (*1*). (B), (H), (N): Mass spectrometric images of m/z 225.171, representing amino-pimonidazole (*4*). (C), (I), (O): Mass spectrometric images of m/z 239.150, representing nitroso-pimonidazole (*2*). (D), (J), (P): Mass spectrometric images of m/z 241.166, representing hydroxylamino-pimonidazole (*3*). (E), (K), (Q): Mass spectrometric images of m/z 530.239, representing the glutathione conjugate of amino-pimonidazole (*6*). (H), (L), (R): pimonidazole immunohistochemical staining.

To evaluate the distributions of pimonidazole and its metabolites, IMS analysis was performed using ultrahigh resolution Fourier transform-ion cyclotron resonance (FTICR)-MS. In addition to pimonidazole and the glutathione conjugate of amino-pimonidazole, peaks with the same m/z as the reduced forms, amino-pimonidazole (see structure *2* in Fig 1), and reductive intermediates of pimonidazole (see structures *3* and *4* in Fig 1) were detected. The m/z values were 225.171 for amino-pimonidazole, 239.150 for nitroso-pimonidazole, and 241.166 for hydroxylamino-pimonidazole (Fig 2B–2D, 2H–2J and 2N–2P). Nitroso- and hydroxylamino-pimonidazole are expected intermediates in the reduction of pimonidazole to amino-pimonidazole. Similar to observations for pimonidazole (Fig 2A, 2G and 2M), the distributions of the reductive metabolites, nitroso-, hydroxylamino- and amino-pimonidazole, were nonspecific in the tumor (Fig 2B–2D, 2H–2J and 2N–2P) and did not correspond to the immunohistochemically positively stained areas for pimonidazole (Fig 2F, 2L and 2R). In contrast, the distribution of the glutathione conjugate of amino-pimonidazole (Fig 2E, 2K and 2Q) correlated with positive pimonidazole immunohistochemical staining from 0.5 h after pimonidazole administration.

To evaluate the effect of the washing of tumor sections on the distribution of the glutathione conjugate of amino-pimonidazole, an IMS analysis was performed on tumor sections from pimonidazole-injected mice with and without washing with hydrophilic solvent (S1 Fig). The ion intensities of the glutathione conjugate of amino-pimonidazole acquired from the IMS images substantially decreased after the washing procedure compared with those of the IMS images without washing.

### Distribution of glutathione in tumors

The distributions of oxidized glutathione and GSH were also evaluated by IMS. The peaks detected in the tissues were assigned using their exact masses. The assignments were verified by structural analysis using CID fragmentation. The peaks generated from the tissue corresponded with those from commercial standards (S1 Table).

**Fig 3:**
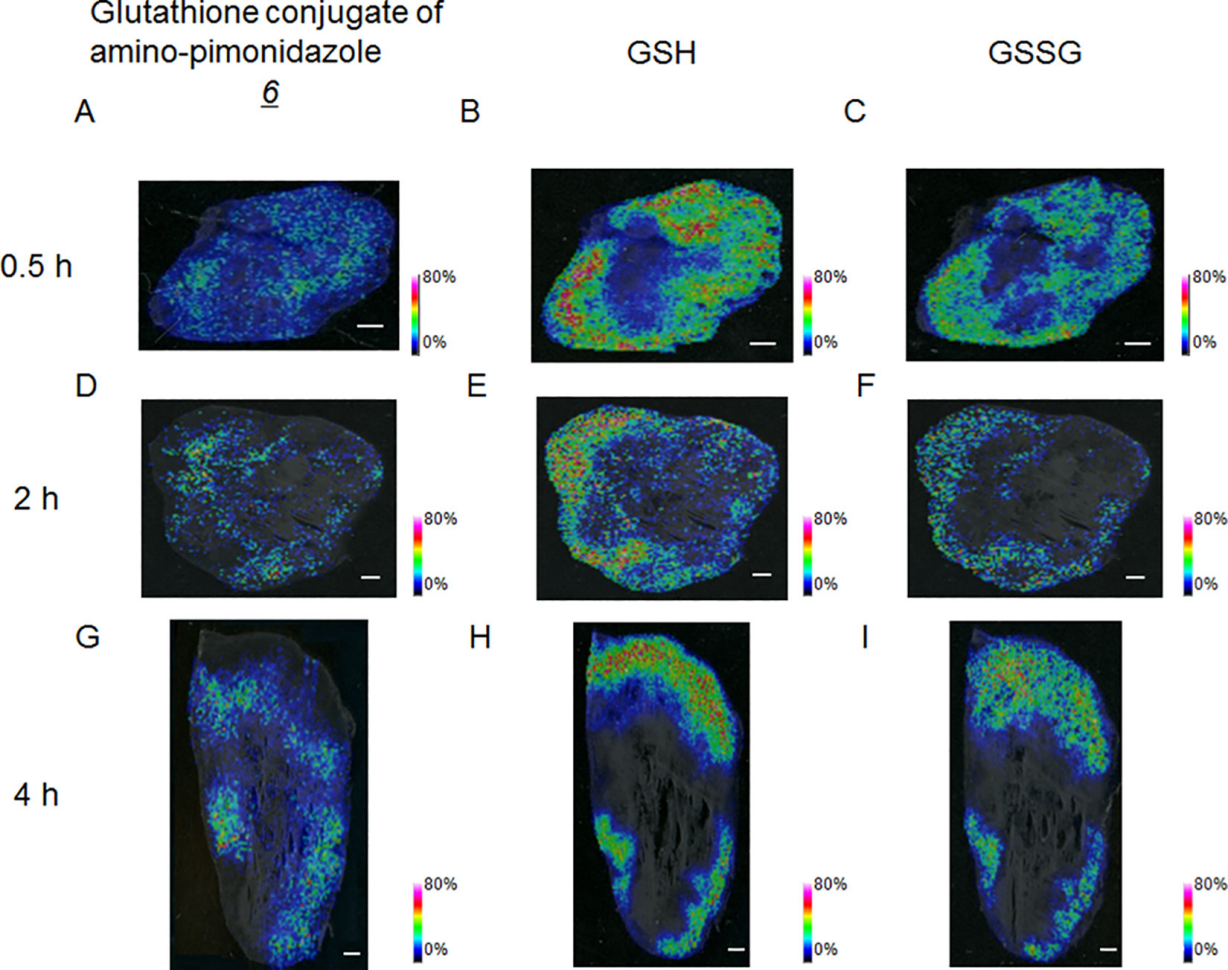
Representative mass spectrometric images of the glutathione conjugate of amino-pimonidazole and oxidized- and reduced glutathione in mouse tumors. The scale bar represents 1 mm. (A)–(C): Mass spectrometric images of mouse tumor 0.5 h after administration of pimonidazole. (D)–(F): Mass spectrometric images of mouse tumor 2 h after administration. (G)–(I): Mass spectrometric images of mouse tumor 4 h after administration. (A), (D), (G): Mass spectrometric images of m/z 530.239, representing the glutathione conjugate of amino-pimonidazole (*6*). (B), (E), (H): Mass spectrometric images of m/z 308.091, representing reduced glutathione (GSH). (C), (F), (I): Mass spectrometric images of m/z 613.159, representing oxidized glutathione (GSSG).

The distribution patterns of GSH (Fig 3B) and the glutathione conjugate of amino-pimonidazole (Fig 3A) did not have an obvious association at an early time point (0.5 h post-injection (p.i.)). At the last timepoint (4 h p.i.), GSH was not detected in the intratumoral area where glutathione conjugate was distributed (Fig 3G and 3H).

### Identification of the glutathione conjugate of amino-pimonidazole in tumors

Synthesized glutathione conjugate of amino-pimonidazole was proposed to consist of two isomers [14]. In LC-MS analysis, the isotope pattern and MS/MS pattern of the synthetic and tumor homogenate samples corresponded (S2 Fig). Thus, the production of the glutathione conjugate of amino-pimonidazole in tumors was verified and the structure was thought to be that of either or both of the two isomers [17].

## Discussion

To elucidate the accumulation mechanism of pimonidazole in hypoxic tumor tissues, we performed an IMS study on tumor sections of pimonidazole-treated mice. We found that the distribution pattern of the glutathione conjugate of amino-pimonidazole (see structure *6* in Fig 1) corresponded to that of the immunohistochemically stained area for pimonidazole, which is considered hypoxic tissue (Fig 2).

The distributions of oxidized- and reduced forms of glutathione were also evaluated by IMS. Glutathione is a ubiquitous tripeptide in mammalian systems. It is usually present in the reduced form (GSH), but it is oxidized and present as a dimer, GSSG, after stimulation, for example in oxidative stress. GSH has the ability to bind to low-molecular-weight compounds to enhance their hydrophilicity, but GSSG does not. Our IMS study showed that the distribution of GSH in the intratumoral area where glutathione conjugate was distributed was attenuated over time (Fig 3). This suggests that the formation of the glutathione conjugate occurred in the tumor tissue. Intracellular GSH depletion has been reported following exposure to 2-nitroimidazole-based agents [18–21]. Thus, it is reasonable to conclude that the heterogeneous absence of GSH within the tumor identified by our IMS study corresponded to the distribution area of the glutathione conjugate of amino-pimonidazole. Furthermore, our previous study revealed that the glutathione conjugate of amino-FMISO was a significant component contributing to the signal in the acquired PET images [13]. We also performed an IMS search enabling simultaneous detection of potential metabolites by broadening the scope of the IMS measurements, comparing mass spectra of tumor sections from FMISO-treated and untreated mice [13]. In that study, we found that no other metabolite except the glutathione conjugate showed the same distribution pattern as the positive areas of immunohistochemical staining. Combined with the observations in the present study, conjugation with GSH is considered to be a common and significant phenomenon when using 2-nitroimidazole-based agents in hypoxic conditions.

2-nitroimidazoles are assumed to bind to thiol groups of macromolecules after reduction of the nitro group of the imidazole ring in a hypoxic environment [22]. Considering that the thiol group of GSH binds to aromatic rings of compounds to form glutathione conjugates, which was confirmed by our LC-MS analysis (S2 Fig), the mechanism of pimonidazole binding to glutathione would be expected to be similar to that of 2-nitroimidazoles covalently binding to macromolecules. In addition, in drug development research, drug candidates are routinely subjected to evaluation for reactive metabolite formation through the glutathione trapping experiment [23], since it is expected that screening for the formation of glutathione conjugates identifies reactive metabolites thought to cause toxicity by covalently modifying macromolecules. Thus, it is reasonable to suggest that pimonidazole is metabolized to the glutathione conjugate, as well as forming adducts to macromolecules, via a reactive intermediate in hypoxic areas of tumors.

To evaluate whether glutathione conjugates of reduced pimonidazole or pimonidazole bound to macromolecules reflected immunohistochemical images of pimonidazole-treated tumors, we performed an IMS study of tumor sections with or without washing by hydrophilic solvents (S1 Fig). During the process of immunohistochemical staining, tissue sections are washed in hydrophilic solvents. The ion intensity of the glutathione conjugate of amino-pimonidazole was decreased markedly by washing, probably because the glutathione conjugates of amino-pimonidazole are relatively hydrophilic and easily washed out by hydrophilic solvents such as water and ethanol. This means that positive staining for pimonidazole could be ascribed to its components being covalently bound to macromolecules, but not to its low-molecular-weight metabolites, including the glutathione conjugate of amino-pimonidazole.

The glutathione conjugate of amino-pimonidazole showed the same distribution pattern as the positive areas of immunohistochemical staining for pimonidazole from the first (0.5 h p.i.) to last timepoints (4 h p.i.) in the experiment. IMS has some advantages over immunohistochemical techniques in that the former can evaluate the distributions of multiple molecules in a single measurement and does not need a specific antibody; therefore, examining the distribution pattern of the glutathione conjugate of amino-pimonidazole by IMS is particularly attractive in evaluating tumor hypoxia using pimonidazole.

In conclusion, our IMS study reveals that pimonidazole accumulates as a glutathione conjugate of reduced pimonidazole (amino-pimonidazole) in hypoxic areas of tumors. This result suggests that 2-nitroimidazole-based compounds are reduced and conjugated with glutathione in hypoxic tumor cells, although it remains unknown why glutathione conjugation was promoted in the hypoxic conditions. Furthermore, our study suggests that IMS provides a novel, effective tool to evaluate tumor hypoxia by visualizing the glutathione conjugate of amino-pimonidazole; this technique can be used in addition to immunohistochemical methods.

## Supporting Information

S1 Fig Representative mass spectrometric images of the glutathione conjugate of amino-pimonidazole with and without washing and pimonidazole immunohistochemical staining in a mouse tumor. The maximum ion intensity was the same in these two mass spectrometric images. (A), (B): Mass spectrometric images of m/z 530.239, representing the glutathione conjugate of amino-pimonidazole (*6*) (A) without and (B) with washing. (C): Pimonidazole immunohistochemical staining.
(TIF)

S2 Fig Validation of the glutathione conjugate of amino-pimonidazole in mouse tumor by isotope pattern and MS/MS analysis. (A): Structure and predicted MS/MS pattern of the glutathione conjugate of amino-pimonidazole. (B): Isotope pattern of the glutathione conjugate of amino-pimonidazole observed from the synthetic form and from that obtained from a mouse tumor. (C): Fragment pattern from MS/MS analysis of ion m/z 530.239 in mouse tumor.
(TIF)

S3 Fig Representative mass spectrometric images of the glutathione conjugate of amino-pimonidazole. (A), (B): Mass spectrometric images of m/z 530.239, representing the glutathione conjugate of amino-pimonidazole (6) acquired from (A) pimonidazole-treated mice or (B) untreated mice. The maximum ion intensity was the same in these two mass spectrometric images.
(TIF)

S1 Table Identification of reduced- and oxidized glutathione (GSH, GSSG) in mouse tumor sections by accurate mass and MS/MS analyses. (DOCX)
