# Supplementary figures and images for: Imaging Mass Spectrometry Revealed the Accumulation Characteristics of the 2-Nitroimidazole-Based Agent “Pimonidazole” in Hypoxia
Source: PLoS One. 2016 Aug 31;11(8):e0161639. doi: 10.1371/journal.pone.0161639 (PMC5007049; doi:10.1371/journal.pone.0161639)

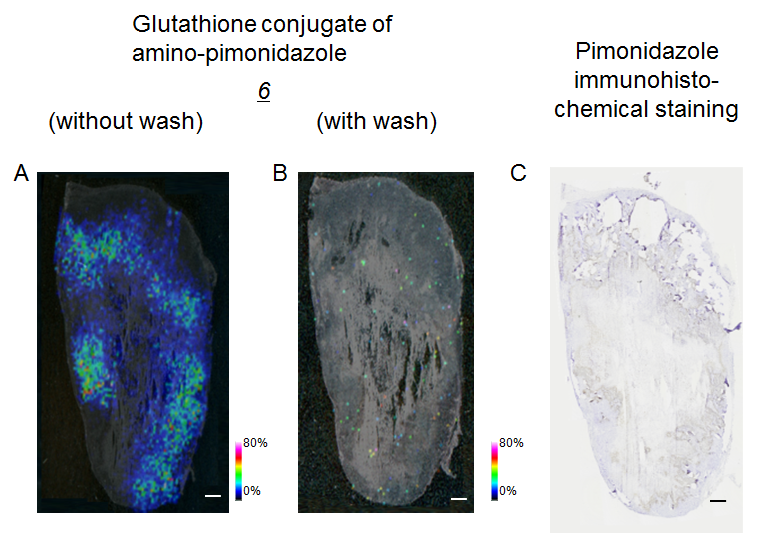

Supplement: S1 Fig — The maximum ion intensity was the same in these two mass spectrometric images. (A), (B): Mass spectrometric images of m/z 530.239, representing the glutathione conjugate of amino-pimonidazole (6) (A) without and (B) with washing. (C): Pimonidazole immunohistochemical staining. (TIF) [file pone.0161639.s001.tif]

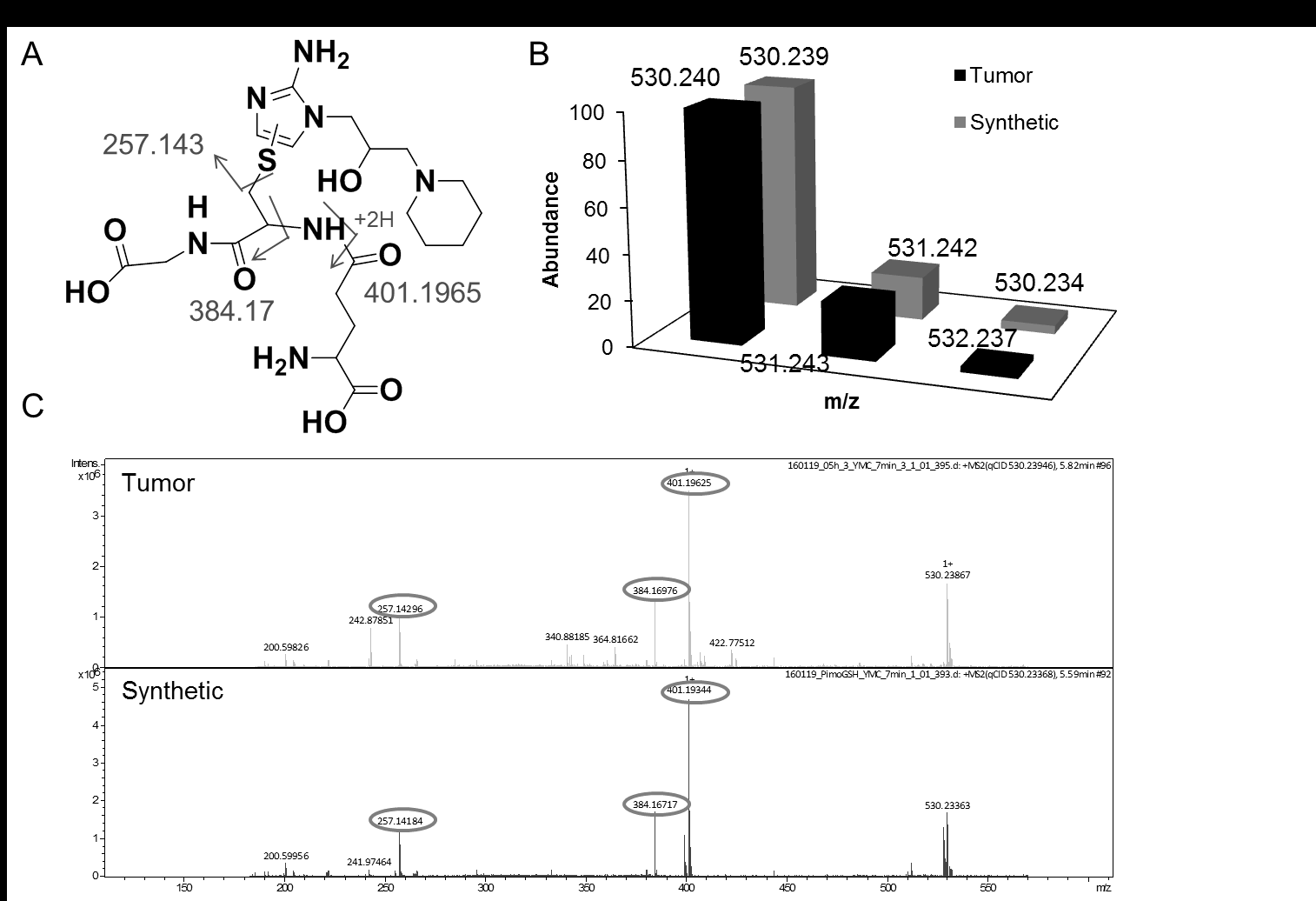

Supplement: S2 Fig — (A): Structure and predicted MS/MS pattern of the glutathione conjugate of amino-pimonidazole. (B): Isotope pattern of the glutathione conjugate of amino-pimonidazole observed from the synthetic form and from that obtained from a mouse tumor. (C): Fragment pattern from MS/MS analysis of ion m/z 530.239 in mouse tumor. (TIF) [file pone.0161639.s002.tif]

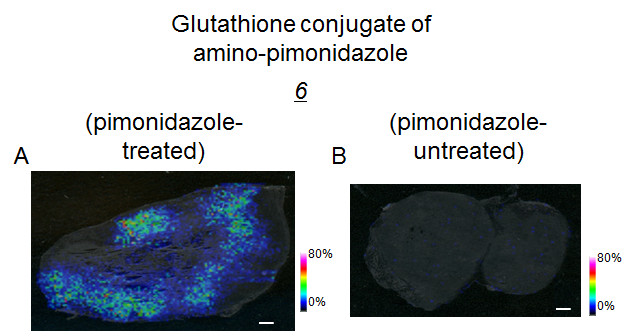

Supplement: S3 Fig — (A), (B): Mass spectrometric images of m/z 530.239, representing the glutathione conjugate of amino-pimonidazole (6) acquired from (A) pimonidazole-treated mice or (B) untreated mice. The maximum ion intensity was the same in these two mass spectrometric images. (TIF) [file pone.0161639.s003.tif]
