# Supplementary material for: Imaging Mass Spectrometry Revealed the Accumulation Characteristics of the 2-Nitroimidazole-Based Agent “Pimonidazole” in Hypoxia
Source: PLoS One. 2016 Aug 31;11(8):e0161639. doi: 10.1371/journal.pone.0161639 (PMC5007049; doi:10.1371/journal.pone.0161639)
Supplement: S1 Table — (DOCX) [file pone.0161639.s004.docx]

**S1 Table. Identification of reduced- and oxidized glutathione (GSH, GSSG) in mouse tumor sections by accurate mass and MS/MS analyses.**

|  | GSH | | GSSG | |
| --- | --- | --- | --- | --- |
|  | m/z | Fragments in MS/MS | m/z | Fragments in MS/MS |
| Tumor | 308.0912 | 130.05013, 162.02225, 179.04883 | 613.1583 | 355.07428, 484.11670 |
| Standard | 308.0922 | 130.05023, 162.02235, 179.04896 | 613.1591 | 355.07466, 484.11727 |
